# Supplementary material for: Are carbon emissions trading and green financial instruments synergistic? -Comprehensive quantitative research based on content analysis
Source: PLoS One. 2024 Mar 7;19(3):e0298601. doi: 10.1371/journal.pone.0298601 (PMC10919601; doi:10.1371/journal.pone.0298601)
Supplement: S1 Data — (ZIP) [file pone.0298601.s001.zip › Data/地级市Z_F工作报告.zip!/╝¬┴╓/│ñ┤║╩╨/└┤╫╘╣1⁄2╓┌║┼í╛┬φ┐╦╩2╛▌═°í┐_2007.docx]

各位代表：

　　我代表市人民政府向大会作工作报告，请予审议，并请列席会议的同志提出意见。

　　一、2006年工作简要回顾

　　2006年，是全面贯彻落实科学发展观，深入实施振兴老工业基地战略，改革开放和现代化建设取得显著成效的一年。一年来，在市委的正确领导下，在市人大、政协的监督和支持下，我们紧紧依靠全市人民，知难而进，开拓创新，较好地完成了市十二届人大四次会议确定的各项目标，实现了“十一五”时期的良好开局。实现地区生产总值1934.1亿元，比上年增长14.5%；实现全口径财政收入210.6亿元，增长14%；完成固定资产投资950.1亿元，增长46.1%；实现城镇居民人均可支配收入11358元，增长12.8%。上述4项主要指标，提前完成本届政府工作目标，标志着我市向富民强市、全面建设小康社会的目标又迈出重要一步。

　　——社会主义新农村建设顺利起步。农业生产喜获丰收,粮食产量达到162.7亿斤。园艺特产之乡发展到20个，无公害和绿色食品基地发展到175万亩，各类优质原料基地发展到530万亩。新建标准化牧业小区100个，养殖大户发展到近3万户，规模饲养的比重达到50%以上。实现农民人均纯收入4480元，增长7.2%。基本实现村村通油（水泥）路，农安、德惠、玉米工业园引水工程，新农村试点的村容镇貌综合整治等一批重点项目全面启动。“三北”防护林建设扎实推进，完成植树造林7800公顷。波罗湖湿地建设步伐加快，生态环境进一步改善。县域经济快速增长，四县（市）和双阳区生产总值、财政收入增幅均高于全市平均水平。

　　——工业经济运行质量明显提高。规模以上工业实现产值2125亿元，增长20.3%；实现利润68.4亿元，增长55.7%。工业经济效益综合指数达到175%，提高19个百分点。高新技术产业产值突破1000亿元，增长22.7%。汽车工业迅速回升，实现产值1475.7亿元，增长21.9%。农产品加工业不断壮大，实现产值294.7亿元，增长24.9%，占全市工业的比重达到13.9%，成为名副其实的支柱产业。光电信息、生物医药、能源、建筑和材料制造等主导产业、重点产业稳步增长，优势正在逐步显现。

　　——现代服务业竞相发展。铁北等物流产业园区稳步发展，物流企业快速壮大，现代物流业营业收入增长22%。旅游和会展业快速发展，被评为“中国最佳旅游城市”试点观察员城市，长影世纪城被评为国家4A级景区，伪满皇宫顺利通过全国首批5A级景区专家初评，汽车文化公园开工建设，成功举办了东博会、农博会、汽配会、冰雪节、电影节、雕塑展、中日韩商务论坛、中日经济合作会议等20个重点展会，旅游和会展业收入分别增长23.2%和21.5%。动漫产业基地初具规模，文化产业迅速发展。房屋交易突破500万平方米，交易额突破百亿元，二手房市场不断完善。浦发银行长春分行正式挂牌运营。市商业银行完成综合治理,资本充足率达到10.29%，不良贷款率降低到1.04%。年末各类金融机构存款余额2396.2亿元、贷款余额2194.8亿元，分别增长17.8%和17.3%。

　　——投资力度进一步加大。固定资产投资净增300亿元,增速在15个副省级城市中位居第1位，前移2位。开工建设项目1994个，其中超亿元255个,新开工1594个。非国有投资592亿元，净增220亿元，占全部投资的66.5%，投资自主增长机制初步形成。整合挖潜存量土地，积极争取项目建设用地，基本保证了项目建设需求。土地投资强度增长25%，土地利用集约化程度明显提高。

　　——各项改革取得突破性进展。国企改革基本结束。列入省市国企改革攻坚计划的254户国有企业，除6户因资产或法律原因外，其余全部完成改革操作程序，建立起新型产权制度，生机与活力明显增强。县(市)、区事业单位人事制度改革全面启动，市属事业单位公开招聘制度全面建立。供暖制度改革实现重大突破，热费补贴由“暗补”变为“明补”。乡镇机构改革、县乡财政管理体制改革和农村义务教育管理体制改革进入收尾阶段。投融资体制改革步伐加快，启动实施项目代建制和重大项目竣工验收、稽查制度，政府投资项目管理进一步规范。人口与计划生育综合改革取得显著成效，文化、医疗卫生体制改革也取得不同程度进展。

　　——对外开放取得突出成绩。新批外商投资企业149家，引进投资超千万美元的外资项目33个、超8000万元的内资项目133个，引进英荷壳牌、美国AB、上海宝钢等一批知名企业，新增世界500强企业投资项目4个。实际利用外资14.1亿美元，引进内资253.8亿元。进出口总额完成52.3亿美元，增长15.1%。汽车及零部件等出口大幅增长，工业制成品出口完成7.5亿元，增长15%，占全部出口的比重达到74%，提高27个百分点。新开通和开发3条国际航线、8条国际中转航线。开发区经济全面提速。生产总值增长26.4%，实际利用外资增长20%，实际利用内资增长35.3%，固定资产投资增长98%，占全市的比重分别达到52.5%、90%、79%和55.5%。引进超千万美元的外资项目19个、超8000万元的内资项目103个,分别占总数的57.6%和79.2%。县域工业集中区引进超千万元的项目274个。

　　——城市建设取得显著成效。市行政中心南迁，南部新城建设拉开序幕。“523台”搬迁,宽城行政中心开工建设，改造大铁北顺利起步。综合科技文化中心前期工作就绪，净月生态城建设快速启动。高新开发区、经济开发区继续保持旺盛的生机与活力，汽车产业开发区以及空港开发区、玉米工业园区开局良好。各城区、开发区以竞相发展的态势，拉开了双中心、多组团的城市发展空间格局。伊通河排污治理工程基本完工，生态工程建设进展顺利，完成三段蓄水。轻轨二期正式通车运营，长双烟铁路完成工程总量的70%， 102国道绕行线等重点项目建设顺利推进，长吉快速铁路、哈大铁路客运专线项目完成前期准备。8路5桥2出口工程竣工，新建道路99条，大中修道路142条，基本完成主次干道改造，道路完好率达到98%。房地产开发施工1214万平方米，增长81.3%。超额完成省下达的棚户区改造任务，拆除棚户区房屋290万平方米，超计划21%。实施货币和房屋安置43334户，安置历年超期回迁居民1596户。改造居民巷道1986条，安装街路照明灯具11128套，三环路以内巷道硬铺装率和亮化率分别达到95%和94%。改造排水管线122公里、供水管线50公里、燃气管线65公里。加大市容环卫投入,更新环卫车辆333辆,水冲公厕达到623座。绿化街路31条，新建大块绿地117块，改造续建4个公园，裕华园开工建设，天嘉公园建成并荣获中国人居环境范例奖，建成区新增绿地300公顷。拆除烟囱327根，治理不合格锅炉501台（套），烟尘排放达标率达到90%，城区优良级天数达到345天，优良率达到94.5%，空气质量进一步提高。城市管理行政执法工作取得明显成效。创建国家卫生城市工作不断深入，顺利通过省级卫生城市验收，具备了申请国家卫生城市的基本条件。

　　——各项社会事业健康发展。积极促进义务教育均衡发展，改造聋哑学校等5所城乡结合部薄弱学校和朝鲜族中学，改造农村中小学危倒校舍11万平方米，农村中小学现代远程教育普及率达到100%。积极推进素质教育，中考推荐生比例提高20个百分点，高考高分段比例提高5个百分点。职业教育迅速发展，毕业生一次性就业率达到95%。城乡实训中心建设初见成效，培训城乡劳动者72.5万人次。科技创新步伐加快，45项光电产业自主创新技术成果实施产业化，76个项目列入省工业企业技术创新工程计划，专利申请量达到2700件，增长15%，技术市场交易额增长30%，承担的国家和省一批重大科技项目成果显著，中俄科技园开工建设。名牌战略扎实推进，皓月、鼎鹿商标荣获中国驰名商标。全面实施人才强市战略，引进各类急需人才1.2万人。公共卫生体系基本建立，农村和城市社区卫生快速发展。北方肝胆医院、儿童医院、妇产医院扩建改造工程竣工，实现了三年改造市属医疗单位的目标。实施食品放心工程卓有成效，在全国31个省会以上城市综合评价检查中位居第四名。人口与计划生育工作不断加强，人口自然增长率低于全省平均水平。和谐文化建设扎实推进，成功开展了“第四届长春文化艺术周”和“2006精品艺术系列演出”活动，演出市场进一步活跃。竞技体育工作取得新成绩。夺取第十五届省运会金牌总数、团体总分等5个第一，成功举办国际滑联短道速滑世界杯赛、瓦萨国际越野滑雪赛等国际大型体育赛事，亚泰足球获中超联赛第四名，华信女足成功冲超。科技拥军工作取得显著成效，预备役炮兵师信息化建设成为全军先进典型。妇女儿童、广播电视、新闻出版、民族宗教、侨务、老龄、残联、气象、人防、震灾、援藏、档案和修志等工作也都取得了新成绩。

　　——举办第六届亚冬会获得圆满成功。亚奥理事会45个成员国和地区全部参会，首次实现亚洲奥林匹克大家庭在冬季运动史上的大团圆。赛会场馆设施先进，各项服务细致周到,竞赛组织规范有序，比赛氛围文明活跃,文化活动异彩纷呈，宣传报道热烈精彩，是亚洲规模最大、最具魅力的冰雪体育盛会。“冰雪亚运、魅力长春”唱响世界，进一步弘扬了奥林匹克精神，提升了长春对外的整体形象和知名度，增强了全市人民的凝聚力和向心力。整个筹备工作所创造的精神和经验，必将成为全市发展各项事业的强大动力和宝贵财富。

　　——社会保障工作扎实推进。开发就业岗位9.7万个，实现就业再就业8.7万人，安置“4050”人员2万人，安置残疾人就业2500人。通过公益性岗位开发，为有就业能力和愿望的“零就业”家庭提供了就业岗位。城镇登记失业率控制在4%以内。启动了新征地农民养老保险试点、城区居民住院医疗保险试点以及榆树、农安新型农村合作医疗试点。全市养老保险参保118.1万人，纳入城镇医疗保障111万人，纳入农村医疗保障182万人，全市失业保险参保66.3万人，工伤保险参保62万人，各项保险基金征缴率达到95%，按时足额发放率达到100%。为32万离退休人员发放养老金26亿元，为29万人次发放失业金6000万元。基本解决改制企业离退休职工养老、医疗保险以及采暖费补贴问题，退休人员社会化管理率达到98.2%。覆盖城乡的社会救助体系基本建立。全市投入救助资金1.9亿元，救助城市低保20余万人。城市低保家庭全部纳入住院保险范围，6315户住房困难家庭享受廉租住房政策，6500户特困家庭享受采暖救助政策，2万余名城乡贫困学生得到教育资助。提前一年完成敬老院三年改造工程，129所农村社会福利中心投入使用，8208名集中供养的五保老人生活质量明显改善。加强社会治安综合治理，深入开展严打斗争，刑事案件立案下降9.6%，破案率提高9.7%，命案破案率达到92.5%。公安后勤保障工作不断提升，新建消防指挥中心和75个派出所。科技强警工作不断加强，提高了打击刑事犯罪和处置突发事件的能力，人民群众安全感进一步增强。进一步落实消防三级管理责任制，加大重大火险整改力度，实现连续4年无重大火灾。深入实施畅通工程，在全国846个参评城市中位居第3名。各类伤亡事故和死亡人数控制在省下达指标以内，安全生产形势稳定。

　　——民主法制和政府自身建设不断加强。自觉接受人大及其常委会的法律监督、工作监督和政协的民主监督，认真办理人大议案、建议和政协提案，办复率达到100%。提请市人大常委会审议通过地方性法规4件，制定政府规章及规范性文件23件。扎实推进依法治市，被评为全国法制宣传教育先进城市。全面履行政府职能，服务型政府建设不断加快。政务中心服务功能进一步强化，依法行政深入推进，电子政务不断发展。深入开展 “清费减负”等多项活动，加快构建“信用长春”，发展环境明显改善。廉政建设和反腐败斗争不断深入，严肃查处各类违法违纪案件，政风行风建设进一步加强。认真办好市长公开电话。进一步落实各级领导干部接待群众来信来访责任制，解决了一大批重大、疑难信访案件，维护了人民群众的合法权益，社会保持和谐稳定。

　　这些成绩的取得，是全面贯彻落实党中央、国务院和省委、省政府各项方针政策的结果，是市委正确领导的结果，是人大代表、政协委员监督与支持的结果，是全市人民团结奋斗和社会各界朋友鼎力相助的结果。在此，我谨代表市人民政府，向辛勤工作在各行各业的全体市民，向给予政府工作积极支持的人大代表和政协委员，向各民主党派、工商联和各界人士，向驻长部队和武警官兵，向所有关心和支持长春改革发展稳定的同志们、朋友们，表示衷心的感谢！

　　过去的一年，我们坚持用科学发展观统领各项工作，着重抓了五个关键环节。一是始终用发展的办法解决前进中的问题，无论遇到什么困难，都坚持加快发展不动摇。二是坚持统筹兼顾，协调推进城乡发展、经济社会发展、人与自然和谐发展，努力使各方面发展相互促进。三是牢固树立以人为本、执政为民的理念，真心实意为群众多办实事、多办好事，努力使改革开放成果惠及千家万户。四是正确处理改革发展稳定的关系，把改革力度、发展速度与社会可承受程度统一起来，在发展中保持社会和谐稳定。五是坚持立足当前与着眼长远相结合，切实解决事关长春长远发展的深层次矛盾和关键问题。

　　在充分肯定所取得成绩的同时，我们也清醒地认识到，当前我市经济社会发展仍面临不少困难和问题。农业基础设施比较薄弱，农村市场发育滞后，农民持续增收的难度加大；经济结构有待进一步优化，转变经济增长方式、提高经济增长质量的任务还相当繁重；产学研结合不够紧密，科技创新和企业自主研发能力较弱；就业再就业任务还很艰巨，部分群众生活困难,社会保障体系还有待进一步完善；土地征用、房屋拆迁等方面还存在一些不容忽视的问题；部分公务员的素质和能力不适应新形势、新任务需要，行政效率和服务水平不高，发展软环境还有待进一步改善，等等。对于这些问题，我们将高度重视，并采取有效措施，认真加以解决。

　　二、2007年主要任务

　　2007年，是保持经济社会发展良好态势，全面完成本届政府任期目标，为长远发展打好基础的关键一年。当前，世界经济正处于新一轮增长期，全国进入消费升级、结构优化新阶段，“南资北移”势头强劲，省委、省政府支持长春在全省率先发展的力度越来越大。实施振兴老工业基地战略以来，我市体制机制创新取得实质性突破，投资拉动效应持续显现，经济增长动力明显增强，具备了进一步加快发展的良好基础和有利条件。只要我们紧紧抓住机遇，强化发展措施，加大工作力度，就一定能够克服前进道路上的各种困难，推动经济社会更好更快发展。

　　2007年政府工作的总体思路是：全面贯彻落实党的十六届四中、五中、六中全会和省委八届十次全会以及市第十一次党代会精神，以邓小平理论和“三个代表”重要思想为指导，以科学发展观为统领，以构建和谐社会为目标，以解决人民群众最关心、最直接、最现实的利益问题为重点，创新发展模式，加快发展速度，提高发展质量，深化改革开放，加大投资力度，加强资源节约和环境保护，增强自主创新能力和产业竞争力，大力发展社会事业，建设和谐文化，完善社会管理，增强社会活力，着力解决民生问题，保持社会和谐稳定，努力实现经济社会更好更快发展。

　　全市经济社会发展的主要预期目标是：地区生产总值增长13%以上，全口径财政收入增长10%左右，固定资产投资增长37%左右，城市居民人均可支配收入增长12%左右，农民人均纯收入增长7%左右，城镇登记失业率控制在4%以内，单位GDP能耗降低7%左右。

　　（一）扎实推进社会主义新农村建设，促进城乡协调发展。坚持以发展农村经济、促进农民持续增收为核心，继续推进农业和农村经济结构战略性调整，加快现代农业建设，大力发展优质粮食生产，集中连片建设优质农产品原料基地，优化种植业结构。突出发展农产品加工业，积极发展精品畜牧业、园艺特产业、绿色食品和劳务经济。建设10大优良畜禽基地，新建标准化牧业小区100个，力争劳务输出100万人次。编制新农村建设总体规划，完成新农村10个试点镇、31个试点村的村容镇貌综合整治。不断完善农村金融服务体系，建立财政支农资金稳定增长机制，扩大公共财政的农村覆盖面,加强农村基础设施建设。加快实施广播电视村村通、乡镇综合文化馆、农村电影放映、农民书屋等工程，完善农村文化基础建设。搞好村镇周边和庭院绿化美化，推进农防林更新改造和近郊生态林等重点工程建设，植树造林5000公顷。改善农村氟病区饮水条件，解决农安、榆树10万人饮水安全问题。新改建乡村公路500公里。

　　（二）加快结构调整，促进工业经济持续快速增长。坚持把工作的着力点放在工业及技术改造上，放在调整结构、转变增长方式、实现可持续发展上。按照走新型工业化道路要求，搭建产业发展平台，加速构建产业集群。加快建设光电产业、生物产业基地，高新技术产品、汽车零部件、医药国家级出口基地，中俄、中德合作基地。培育壮大汽车、玉米、轨道客车产业园区。实施名牌战略，争创国家名牌产品和驰名商标。加快新产品开发，提升产品市场竞争力。继续举全市之力支持一汽加快发展，扶持大成、德大、皓月、富奥、西门子VDO等重点企业做大做强，加快培育一批主业突出、竞争力强的中小企业，规模以上工业企业突破1000户。加强工业经济运行监控和服务，搞好资金、电力、燃气保障，支持重点企业、重点产品增加生产，提高效益。规模以上工业产值增长16%左右。

　　（三）努力扩大消费，加快发展现代服务业。改造长春铁路货运口岸，加快建设重点物流园区，引进和培育物流骨干企业，物流业营业收入增长20%以上。抓好旅游重点项目建设，加大旅游促销力度，争创“中国最佳绿色旅游城市”，旅游业收入增长20%以上。大力发展会展业，办好冰雪节、汽车节、汽博会、东博会等重点展会，举办各类会展活动125项以上。集中建设长江路、重庆路、桂林路、红旗街、东盛路等重点商业街区，继续推广连锁经营、物流配送等现代流通方式。引导住宅类房地产优化供给结构，扩大中低档商品房建设，搞好居民回迁房建设,大力激活二手房市场，促进房地产市场健康发展。加快发展地方金融机构，推进社会信用体系建设，做大做强融资担保机构。继续扩大汽车、旅游、信息、健身等消费,促进消费结构升级。积极发展农村新型流通组织，不断开拓农村消费市场。整顿和规范市场秩序，改善消费环境，保护消费者利益。社会消费品零售总额增长12%左右，第三产业增加值增长13%左右。

　　（四）深入开展“项目建设年”活动，突出抓好重点项目建设。发挥企业投资主体作用，加大政府推动力度，充分挖掘各方面潜力，广泛吸引社会资本，集中全社会力量抓投入、上项目，促进投资主体多元化、资金来源社会化，努力实现民间投资、外企投资、政府投资的新突破。完成固定资产投资1300亿元，净增350亿元左右。围绕“十一五”规划，谋划启动一批关系全局、带动作用强、发展后劲足的大项目，突出抓好主导产业、城市建设、农业水利、公益事业等骨干和重点项目。积极推进SUV和皮卡20万辆汽车、100万吨化工醇、100万张皮革深加工、PLED有机电致发光屏、奇健生物制药、国家生物产业基地实验动物中心、九台华能电厂、壳牌油母页岩、亚泰水泥熟料等一批市场前景好、技术含量高、经济效益显著的骨干项目。延伸项目集中审批“绿色通道”，强化重大项目推进服务，构筑更具吸引力的投资环境。节约利用土地，盘活土地存量资源，确保重点项目需求。

　　（五）继续深化改革、扩大开放，进一步增强经济社会发展的动力和活力。巩固国企改革成果，积极稳妥处理各种遗留问题，支持改制企业加快发展。完善国有资产监督管理体制，加大监管力度，确保国有资产保值增值。力争完成厂办大集体改革试点。积极推进文化体制改革。进一步深化事业单位人事制度改革，扩大面向社会公开招聘范围，促进从身份管理向岗位管理转变。全面实施民营经济腾飞计划，推动民营经济大发展。以发展中小型企业为重点，实施创业孵化基地工程。搭建银企对接平台，着力解决中小企业融资难问题。大力发展和扶持个体工商户。进一步完善支持民营经济发展的配套政策，降低门槛、减轻负担、鼓励创业、发展企业，努力营造有利于全民创业的浓厚氛围和优良环境。

　　改善招商基础，健全招商体系，拓展招商领域，突出招商重点，创新招商方式，积极引进境内外资金、先进技术、先进管理和高端人才，大力发展以优势产业的重大项目为龙头的产业集群，努力实现招商规模和质量的新突破，实际利用内、外资均增长20%以上。巩固日本、韩国、香港等周边传统市场，深度开发美、德、英、法、意等欧美市场，积极开发东南亚和台湾地区市场，进出口总额完成56亿美元以上。不断优化出口产品结构，工业制成品出口比重达到80%以上，增长20%以上。积极鼓励企业“走出去”，充分利用国际国内两种资源、两个市场。

　　加快开发区建设。加强和完善基础设施配套建设，提高项目建设的承载能力。深化体制机制创新，优化投资环境，发挥政策优势，形成集聚效应，不断增强对外开放的先导和辐射带动作用。高新、经开、净月、汽车四个开发区完成固定资产投资609亿元以上,其中工业投资占60%以上;生产总值和一般预算全口径财政收入均增长20%以上。加快建设五棵树、合隆、米沙子、奢岭、卡伦等县域工业集中区。

　　（六）加强创新型城市建设，不断增强可持续发展能力。积极实施《长春市中长期科技发展规划纲要》。加强政府引导，加大政府科技投入,加快推进以企业为主体的自主创新体系建设，不断提高自主创新能力，促进高新技术成果的开发、转化和产业化，高新技术产业产值增长20%左右。围绕重点领域、关键技术，适时启动12个重大技术专项，增强原始创新、集成创新和引进消化吸收再创新能力。实施“科技成果转化百亿增值工程”和“科技创业风险投资促进百户企业快速发展工程”，为科技成果转化和科技企业发展提供全方位政策支持。加强知识产权创造和保护。强化科技成果转化平台建设，逐步培育建设一批国家级企业技术中心和科技企业孵化器。

　　进一步强化环境综合治理和自然生态保护。以解决制约经济社会发展和危害群众健康安全的环境问题为重点，加快建设资源节约型、环境友好型社会。深入落实环保目标责任制，强化环保政绩考核及违法违纪责任追究。依靠科技进步，发展循环经济，推广清洁生产，加大执法力度，倡导生态文明，努力改变先污染后治理的状况。加强集中式饮用水源安全保护和重点流域污染防控, 取缔一级保护区内排污企业，关闭二级保护区直接排污口。深入开展“整治违法排污企业保障群众健康环保专项行动”，着力整治扰民环境污染，依法淘汰落后工艺和落后产品，努力改善生态环境质量，维护环境健康安全，切实增强可持续发展能力。

　　（七）加强城市建设与管理，全力创建国家卫生城市。坚持统筹兼顾、循序渐进原则，协调推进南部新城、净月生态城建设和铁北改造，加快推进城市综合换乘中心、铁路西客站项目建设前期准备，力争开工建设长吉城际铁路、哈大铁路客运专线、长松高速公路、轻轨4号线。实施102国道绕行线、长乐公路出入口等10路4桥2出口建设、改造工程。改造供水管网20公里，发展燃气用户2万户，新增集中供热面积200万平方米。

　　对照《国家卫生城市标准》，加大创建工作力度，逐项整改达标，力争通过国家考核验收。继续改造棚户区，拆除房屋265万平方米以上，居民安置回迁率达到80%以上。房地产开发施工800万平方米、竣工270万平方米以上。继续整治裸露地面，实施居民巷道、街路亮化、排水明沟改造。新增绿化街路25条，新建大块绿地142块。基本完成裕华园改造。加强城市管理综合行政执法，进一步落实“门前三包”责任制，深入搞好市容环境综合整治，查处扰民污染。加大城区物业治理力度,提高物业管理水平。继续建设三间村粪便无害化处理厂、蘑菇屯垃圾无害化处理场、南部污水处理厂，通过北郊污水处理厂升级改造等措施，污水二级处理率达到70%以上。

　　（八）大力发展社会事业，促进社会和谐发展。深入开展群众性精神文明创建活动，在全社会树立以“八荣八耻”为主要内容的社会主义荣辱观，形成知荣辱、讲正气、促和谐的良好风尚。坚持教育优先发展，促进教育公平。继续加大对教育事业的投入。巩固义务教育成果，继续实施薄弱校改造工程，在铁北建设一所省级示范高中。全力支持农村教育，逐步缩小城乡教育发展差距，促进义务教育均衡发展。全部免除农村义务教育阶段学生学杂费，对贫困家庭学生免费提供教科书并补助寄宿生生活费，着力解决城市低收入家庭和农民工子女义务教育阶段上学困难问题，确保每个孩子都有接受义务教育的机会。大力发展职业教育，加快产业技能人才培训中心和县（市）职教中心建设，强化农村实用技术培训，完成各类培训80万人次。建立一批标准化、示范性“农民夜校”和社区活动阵地。鼓励、支持、规范、引导民办教育健康发展。全力支持高校加快发展。实施人才强市战略，大力开发人才资源，切实强化高层次和紧缺人才队伍建设，引进高层次及各类急需紧缺人才1万人左右。全面落实《全民科学素质行动计划纲要》，努力提高市民科学素质。

　　强化政府公共卫生责任，完善城乡医疗卫生服务体系，建立医疗保障体系。加快城市社区卫生事业发展，强化医疗行业监管和医疗卫生服务，推进医药分业改革试点，提高医疗服务质量，减轻患者就医负担。进一步扩大城区住院医疗保险范围。启动德惠市、双阳区和城区新型农村合作医疗试点，实现农村人口医疗保障制度全覆盖。继续推进食品放心工程建设，加强药品监管和农村医药“三网”建设，整顿规范药品生产和流通秩序，保障群众饮食用药安全。稳定人口低生育水平，提高出生人口素质。

　　加快发展文化事业，加强公益性文化设施建设，逐步完善公共文化服务体系，不断满足人民群众需求。大力发展文化产业，鼓励非公有资本依法进入文化产业。抓好重大文化产业项目，确保综合文化科技中心按期开工建设。加快体育产业化进程，办好越野滑雪世界杯赛、亚洲冰球联赛、全国篮球甲级联赛等国际国内体育赛事，促进体育事业健康发展。繁荣文学艺术、社会科学、广播影视、新闻出版事业。

　　（九）不断完善城乡就业和社会保障体系，切实解决涉及群众切身利益的问题。坚持以人为本，关注民生，努力构建和谐社会。大力发展劳动密集型产业、服务业，多渠道、多方式增加就业岗位。加强政府对就业工作的指导和服务，全面落实促进就业再就业的各项政策，按照国家城乡统筹就业试点部署，进一步构建制度健全、功能完善、规范高效、服务社会、覆盖城乡的就业服务体系。新增就业岗位10万个,安置下岗失业人员7万人，其中“4050”人员1万人。建立“零就业”家庭动态援助机制，切实解决“零就业”家庭就业问题。培训下岗失业人员2.7万人，培训后就业率达到65%以上。健全劳动保障监察体制和劳动争议调处机制，维护劳动者特别是农民工的合法权益。

　　立足于全体社会成员普遍受惠，多渠道筹集社会保障基金，适度增加财政的社会保障投入。进一步调整社会保险参保结构，扩大失地农民养老保险、城区居民住院医疗保险覆盖面。积极探索建立农村养老保险制度，启动机关事业单位养老保险改革试点，推进非公经济组织和个体户参保，逐步将符合条件的城镇各类从业人员纳入社会保险范围，全市养老保险参保130万人以上，失业保险参保76万人以上，城镇医疗保险参保120万人以上，全市工伤保险参保65万人以上，生育保险参保28万人以上。加快信息和服务网络建设，全面提高退休人员社会化管理服务水平。继续实施扶残助业工程，适时提高城市最低保障标准。不断完善城乡低保、农村五保户供养、特困户救助、灾民救助等制度，加强法律援助，逐步建立社会保险、社会救助、社会福利、慈善事业相互衔接的社会保障体系。继续开展以科技拥军为特色的“双拥”工作，争创国家双拥模范城。广泛开展创建和谐社区活动。深入创建“平安长春”。严厉打击各种刑事犯罪，强化社会治安综合治理，搭建社区、农村、厂区警务平台，整合、建设安全防控网络体系，开展打击黄赌毒斗争，不断增强人民群众安全感。进一步加强信访工作，及时化解各类矛盾。切实加强生产安全、交通安全和消防安全管理，预防重特大事故发生。注重城市公共安全，完善应急管理体制机制，有效应对各种风险。加强国家安全工作，严密防范、坚决打击各种敌对势力的渗透和破坏活动。

　　（十）加强民主法制建设，努力建设服务型政府。深入贯彻《各级人民代表大会常务委员会监督法》，认真执行人大及其常委会决议，完善工作报告制度，自觉接受人大的法律监督、工作监督。及时向市政协通报重要工作，更好地尊重和发挥人民政协政治协商、民主监督、参政议政职能。大力推进民主政治，扩大基层民主，保证人民群众当家作主、依法行使民主权利。健全社会组织，完善居（村）民自治，依法组织好社区居委会、村委会换届选举。全面落实“五.五”普法规划。

　　进一步优化经济发展软环境，加快社会信用体系建设。强化政府社会管理和公共服务职能，创新服务体制，改进服务方式，提高服务质量，逐步形成惠及全民的基本公共服务体系。继续贯彻《依法行政实施纲要》，推进法治政府建设，加快推行行政执法责任制。加大政务公开力度，进一步提高政务中心行政审批即办件的比重。深化信息公开，提高行政运行透明度。完善政府新闻发布制度，满足社会公众对政府信息的需求。加快发展电子政务，提高行政运行效率和服务公众能力。加强和改进审计工作。认真落实党风廉政建设责任制，严格履行“一岗双责”制。深入治理商业贿赂,严格执行建设工程招投标、土地招拍挂、政府采购和产权交易制度，推行项目代建制。大力改进政风，继续开展民主评议政行风和“百名处长”活动，严肃查处干扰和破坏发展软环境的重大案件。认真贯彻《公务员法》，着力提高促进和服务发展的能力，努力建设一支政治坚定、业务精通、清正廉洁、作风优良的公务员队伍。

　　各位代表，回首过去的一年，我们用辛勤的劳动和汗水，换来了丰硕的果实；展望新的一年，我们面临着新的机遇和挑战。让我们紧密团结在以胡锦涛同志为总书记的党中央周围，在市委的领导下，团结和带领全市各族人民，齐心协力，开拓进取，扎实工作，为实现更好更快发展和全面建设小康社会的宏伟目标而努力奋斗！
